# Chronic Migraine Pathophysiology and Treatment: A Review of Current Perspectives

**DOI:** 10.3389/fpain.2021.705276

**Published:** 2021-08-25

**Authors:** Tiffani J. Mungoven, Luke A. Henderson, Noemi Meylakh

**Affiliations:** Department of Anatomy and Histology, Brain and Mind Centre, University of Sydney, Sydney, NSW, Australia

**Keywords:** trigeminal nerve, functional connectivity, CGRP, hypothalamus, periaqueductal gray, spinal trigeminal nucleus

## Abstract

Chronic migraine is a disabling neurological disorder that imposes a considerable burden on individual and socioeconomic outcomes. Chronic migraine is defined as headaches occurring on at least 15 days per month with at least eight of these fulfilling the criteria for migraine. Chronic migraine typically evolves from episodic migraine as a result of increasing attack frequency and/or several other risk factors that have been implicated with migraine chronification. Despite this evolution, chronic migraine likely develops into its own distinct clinical entity, with unique features and pathophysiology separating it from episodic migraine. Furthermore, chronic migraine is characterized with higher disability and incidence of comorbidities in comparison to episodic migraine. While existing migraine studies primarily focus on episodic migraine, less is known about chronic migraine pathophysiology. Mounting evidence on aberrant alterations suggest that pronounced functional and structural brain changes, central sensitization and neuroinflammation may underlie chronic migraine mechanisms. Current treatment options for chronic migraine include risk factor modification, acute and prophylactic therapies, evidence-based treatments such as onabotulinumtoxinA, topiramate and newly approved calcitonin gene-related peptide or receptor targeted monoclonal antibodies. Unfortunately, treatments are still predominantly ineffective in aborting migraine attacks and decreasing intensity and frequency, and poor adherence and compliance with preventative medications remains a significant challenge. Novel emerging chronic migraine treatments such as neuromodulation offer promising therapeutic approaches that warrant further investigation. The aim of this narrative review is to provide an update of current knowledge and perspectives regarding chronic migraine background, pathophysiology, current and emerging treatment options with the intention of facilitating future research into this debilitating and largely indeterminant disorder.

## Introduction

Chronic migraine is a debilitative neurological disorder that impacts on average 1.4–2.2% of the global population and imposes a significant individual and socioeconomic burden (1). With a biased prevalence toward women (1.7–4.0%) compared to men (0.6–0.7%) (1), chronic migraine incidence peaks during midlife, affecting the most productive years of an individual's life (2). Direct medical costs of chronic migraine including pharmacological treatments, diagnostic tests, emergency department visits and hospitalizations are three times higher on average than the episodic migraine annual burden in the United States (3), Europe (4) and Australia (5). In Australia, chronic migraine incurred a total annual economic cost of $8.1 billion, with total health system costs of $2.8 billion, productivity costs of $4.1 billion, career costs of $84 million and well-being costs of $13.5 billion (5). Chronic migraine typically evolves from episodic migraine with an annual progression rate of ~3% (6), suggesting chronic migraine may be a progressive neurological disorder. While chronic migraine and episodic migraine are often conceptualized as disorders of the same spectrum, some researchers and clinicians suggest that chronic migraine develops into its own distinct clinical entity with an episodic migraine predisposition, accounting for ~8% of the total migraine population (7–10). Chronic migraine imparts a significantly greater burden than episodic migraine with disability scores reported to be nearly twice as high (11).

A migraine is characterized by moderate to severe attacks of unilateral pulsating head pain, associated with photophobia, phonophobia, nausea and/or vomiting, typically lasting 4–72 h (12). An individual with chronic migraine must have headaches on at least 15 days per month for at least three months, with at least eight of these headaches satisfying criteria of migraine headache (12). As a consequence, episodic migraine is distinguishable from chronic migraine by reduced headache/migraine frequency and commonly involves ~1–2 migraines/headaches per month. Classically, the migraine attack is comprised of three phases: a premonitory phase, the migraine headache itself, and a postdrome phase. The premonitory phase occurs 24–48 h prior to the headache phase and is typically characterized by symptoms such as mood alterations, fatigue and neck discomfort (13). In addition, approximately one third of migraineurs experience an aura, comprised of transient focal neurological symptoms of visual, sensory or motor disturbances, which may occur simultaneously with the premonitory or migraine headache phases (14, 15). The headache phase is followed by the postdrome phase, which lasts for 72 h and is characterized by non-headache symptoms including tiredness, difficulties concentrating and a stiff neck (16). The pain- and symptom- free interictal period between migraine attacks may vary in duration depending on the migraineurs' chronicity. Given the high attack frequency and symptom severity of chronic migraine, it is likely that sufferers are persistently in a migraine headache and/or premonitory-like phase with limited neurological recovery and baseline restoration between attacks.

While the mechanisms underlying the transformation from episodic to chronic migraine appear to be complex, risk factors for progression include high baseline episodic migraine attack frequency, acute medication overuse, obesity, stressful life events, female gender and lower socioeconomic status (10, 17–19). Additionally, chronic migraineurs have an increased incidence of comorbid psychiatric disorders such as anxiety and depression (8, 20–22). These conditions perpetuate and exacerbate migraine frequency and intensity, exhibiting a bidirectional relationship (23), and highlight the multifaceted nature of the chronic migraine condition (24). Risk factor modification through appraisal is essential for headache management and a greater understanding of migraine chronification may further aid in the development of selective therapeutics to facilitate the effective treatment and management of chronic migraine. Current episodic migraine treatments are often ineffective in chronic migraineurs and may further perpetuate migraine attack intensity and frequency (10). The focus of this review is to describe what is currently known about chronic migraine with a focus on pathophysiological mechanisms as well as acute and prophylactic treatment options with consideration of emerging therapeutic strategies to treat chronic migraine.

## Pathophysiology of Chronic Migraine

**Figure 1:**
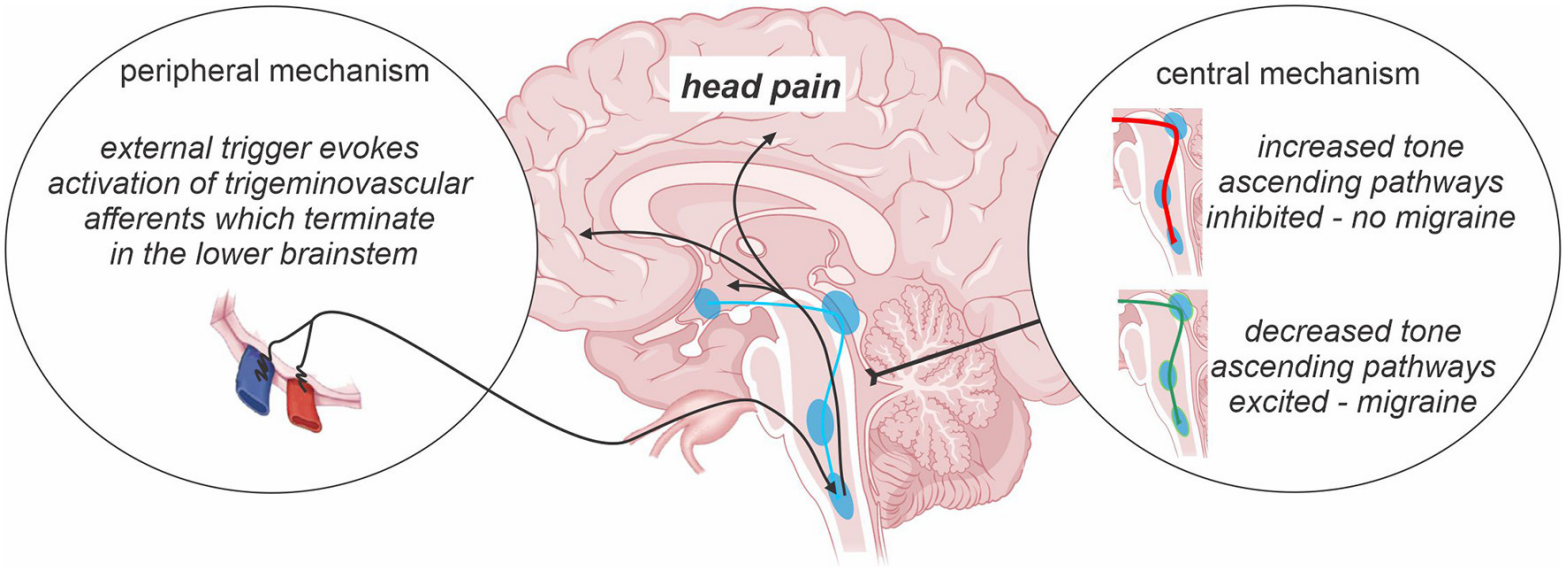
Central vs. peripheral mechanism of migraine. The prevailing theories of migraine initiation include the existence of a peripheral trigger and/or central nervous system changes including oscillations in the sensitivity of descending pain modulatory pathways across the migraine cycle. Such changes in brainstem “tone” are proposed to either prevent or allow an external trigger or basal brainstem activity from evoking activity changes in ascending pathways which are ultimately responsible for the presence of a migraine event.

The underlying pathophysiology of chronic migraine is poorly understood and remains an evolving area of research. Whilst the development of migraine theories has evolved over time without consensus as to its pathophysiology, there are currently two major schools of thought with regards to the underlying mechanism of migraine in general—one which suggests that migraines are generated by external triggers and another which suggest that migraines are largely generated from changes within the brain itself (Figure 1). The early vascular theory of migraine stemmed from the hypothesis that initiation of migraine attacks occurred via activation of perivascular nerves innervating major cerebral vessels (25). Whilst cerebrovascular changes were for some time considered the foundation of migraine pathophysiology, recent evidence has emerged of a critical role for neural changes in subcortical sites in migraine generation (26, 27). Such evidence includes observations that symptoms including tiredness and reduced concentration occur hours before the migraine onset (28) and experimental animal evidence that activation of brainstem trigeminovascular neurons by cortical spreading depression can occur independently of peripheral input (29). It has been suggested that migraine is a dysfunction of subcortical sites below the level of the diencephalon, which result in an “abnormal perception of basal level of primary traffic” (30). Indeed, it was recently proposed that brainstem activity oscillates between enhanced, threshold and diminished neural “tone” states (31). When the brainstem is in a state of diminished tone, on-going endogenous analgesic circuits are ineffective at modulating incoming noxious inputs and an external trigger can activate trigeminal pathways and evoke head pain; vice versa when brainstem tone is enhanced. There is also a suggestion that the hypothalamic function is critical and may be involved in altering the sensitivity of brainstem sites that themselves modulate incoming trigeminal inputs (32, 33). It appears that migraine is a complex disorder with a synergistic relationship between peripheral and central nervous systems, which together are involved in migraine generation (34). Whilst it may be the case that peripheral and central mechanisms co-exist, the balance between these drivers may differ between individual migraineurs. Furthermore, it is likely that the contribution of central and peripheral mechanisms to migraine generation alter when an individual transitions from episodic to chronic migraine or that they simply change over time. Whilst our understanding is limited, it is likely that significant similarities exist between the mechanisms responsible for chronic migraine, episodic migraine and other chronic orofacial pain conditions. Structural and functional brain changes in pain-related regions, atypical pain processing, cortical hyperexcitability, central sensitization and neurogenic inflammation may all play a role in chronic migraine transformation, initiation and maintenance.

### Medication Overuse in Headache Chronification

One of the difficulties in chronic migraine diagnosis and assessing the underlying pathophysiology is that its characteristics can easily overlap with that of medication overuse headache disorder (MOH). MOH is characterized by headaches on at least 15 days a month, occurs within the framework of an existing migraine or tension type headache disorder, and results from an individual taking analgesic medications to treat the original headache condition (35). Evidence shows that the overuse of triptans, opioids and combination analgesics result in headache chronification more rapidly than the overuse of simple analgesics to treat migraine (36). The risk of developing MOH also increases in individuals with a family history of MOH or substance overuse (37), and migraine is an underlying disorder in around 60–80% of patients, suggesting that inheritability and medication specific mechanisms may be involved (35, 38, 39). A recent preclinical model of MOH found that persistent exposure to 5-HT_1F_ receptor agonists and not CGRP receptor antagonists exhibited a potential risk of inducing MOH (40). More specifically, 5-HT_1F_ receptor agonists resulted in sensitization and neuroplastic alterations of trigeminal sensory afferents similar to that of sumatriptan. This finding suggests that 5-HT_1F_ receptor agonists reflect a similar MOH-risk profile to the related 5-HT_1B/1D_ receptor agonist triptan drugs, highlighting the importance of mitigating the increased risk of MOH development.

Whilst some authors suggest that pathophysiological mechanisms involved in migraine are also present in MOH (35), emerging evidence has begun to provide insights into potential underlying mechanisms of medication overuse in headache chronification (41–43). For example, comparison of chronic migraineurs with and without MOH revealed significant differences in gray matter volumes in the orbitofrontal and parahippocampal regions (42). Another study found that individuals with MOH displayed greater volume in brain areas related to pain processing and antinociception such as the midbrain periaqueductal gray matter (PAG) and decreased volume in the orbitofrontal cortex (44). Critically, the authors showed that following 12 months of “detoxification” the volume of the PAG returned to control levels only in those whose headache frequency reduced (45), implying that the volumetric changes in some regions are reversible and related to medication overuse. It appears that a strong connection between headache specific pain pathways and effects of excessive medication on the descending modulation of the pathways exist and underpin the development of MOH from most commonly a migraine condition (46). Given that MOH emerges most frequently in individuals with an underlying migraine condition, it is likely that the overall mechanisms responsible for chronic migraine and MOH overlap to some degree. More studies are needed to unravel the potential subtle differences between these conditions, which itself may provide a biological marker for better diagnosis and treatment.

### Sensitization of the Trigeminal System and Neurogenic Inflammation

It has been suggested that sensitization of trigeminal afferents is critical for the development of chronic migraine. It is known that chronic migraineurs express significant increases of transient receptor potential vanilloid type-1 receptor (TRPV1) immunoreactive in nerve fibers innervating the walls of scalp arteries (47). Expressed in small diameter sensory neurons, TRPV1 receptors promote excitation of the trigeminovascular pathway and mediate the release of calcitonin gene related peptide (CGRP) and substance P leading to sensitization (48, 49). It was reported that the efficacy of onabotulinumtoxinA injections is predicted by elevated levels of CGRP and vasoactive intestinal peptide (VIP) (50), which leads to a reduction of TRPV1-positive neurons in the trigeminal ganglion of rats following an injection (51). This process occurs through the inhibition of TRPV1 plasma membrane trafficking and proteasome-mediated degradation in the cytoplasm (51), overall suggestive of the possibility that peripheral sensitization is a key feature of chronic migraine. Chronic migraineurs exhibit higher interictal plasma levels of vasoactive neuropeptides, such as CGRP, VIP and pituitary adenylate cyclase activating polypeptide-38 (PACAP-38) compared to episodic migraineurs (52–55), which is consistent with altered trigeminal nerve function and indicates that these neuropeptides may be key mediators of neurogenic inflammation.

PACAP-38 and VIP are both vasoactive peptides belonging to the secretin/glucagon peptide superfamily and are found in perivascular parasympathetic nerve fibers (56, 57). PACAP-38 has widespread expression in headache regulatory regions such as the pituitary, brainstem, hypothalamus and cortex (58), and clinical studies have revealed that infusion of PACAP-38 produces migraine in 73% of migraineurs without aura subjects (59). In contrast, VIP-induced vasodilation of the cranial arteries failed to trigger migraine attacks in patients with migraine (60) and only produced a mild, short lasting headache in healthy subjects (61). PACAP-38 infusion was also found to elicit photophobia, meningeal dilation and elevate neural activation in the trigeminal ganglia and spinal trigeminal nucleus (SpV) in wild-type mice compared to PACAP-38 deficient mice (62). These studies support a role for PACAP-38 in migraine pathophysiology in which a persistent expression may underlie chronic migraine events. In addition, emerging evidence suggests that adenosine 5′-triphosphate-sensitive potassium (K_ATP_) channel openers may play a role in mechanisms of migraine through its vasodilatory expression in cranial arteries, trigeminal ganglion and SpV (63, 64). In clinical studies, intravenous infusion of synthetic K_ATP_ channel opener, levcromakalim dilated extracerebral arteries and induced headache in non-migraine subjects (65) while migraine attacks were generated in migraine subjects with (66) and without aura (67). This suggests that opening of K_ATP_ channels may potentially be a novel signaling pathway that requires further investigation in the context of migraine pathophysiology.

There is some evidence that the trigeminal nerve is structurally altered in episodic migraine with histological evidence of altered fiber arrangement and magnetic resonance imaging (MRI) data showing altered trigeminal nerve volume and free water diffusivity, consistent with altered anatomical fiber arrangement (68). Whilst no study has explored trigeminal nerve anatomy in chronic migraineurs, we know that similar MRI changes occur in the trigeminal nerve of other forms of chronic orofacial pain (69), which suggests that similar changes in nerve structure may occur in individuals with chronic migraine. Mounting evidence suggests that migraine is comorbid with orofacial pain conditions such as temporomandibular disorders (TMD) (70, 71), which demonstrates that similar underlying pathophysiological mechanisms may be apparent. Indeed, nearly two-thirds of all individuals with chronic daily headaches had concurrent TMD (72) and the association between painful TMDs and headache was reported to be highest in chronic migraine (73). Recent preclinical studies found that pre-existing myogenic TMD can upregulate nitroglycerin-induced CGRP in SpV and enhance migraine-like hypersensitivity (74). In addition, it has been shown that both episodic migraine and painful trigeminal neuropathy are associated with alterations in very slow resting neural oscillations, and it has been proposed that altered glial-neural interactions may underpin these similar neural pattern changes (75, 76). Whilst the overlap between migraine and other forms of orofacial chronic pain are yet to be defined, preliminary evidence suggests the possibility that mechanistic overlaps may be present and require further investigation.

**Figure 2:**
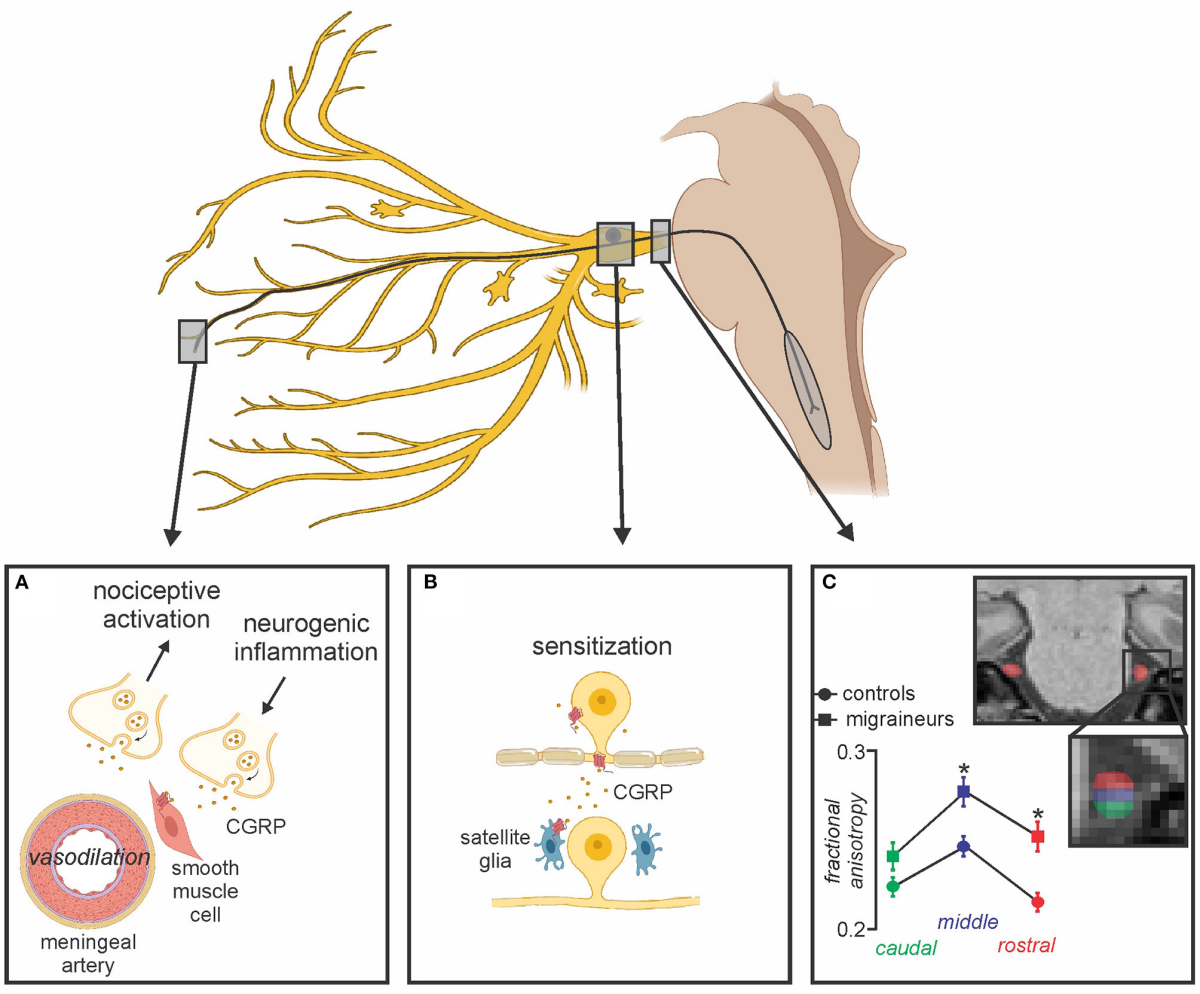
Peripheral changes associated with migraine. **(A)** CGRP released from peripheral terminals onto receptors act on smooth muscle cells leading to potent vasodilation of meningeal arteries, subsequent neurogenic inflammation, activation of meningeal nociceptors and peripheral sensitization; **(B)** Secreted CGRP from the trigeminal ganglion interacts with adjacent CGRP receptors on satellite glia resulting in prolonged sensitization; **(C)** Alterations in trigeminal nerve volume and free water diffusivity evident in episodic migraineurs compared to healthy controls may also be associated with chronic migraine. Modified from (68).

Additionally, there are also processes in the trigeminal ganglion itself that may be involved in chronic migraine. For example, it has been shown that CGRPergic nociceptor neurons can secrete CGRP in the trigeminal ganglion which can then interact with CGRP receptors on satellite glia to release nitric oxide. This release can then enhance neural activity and may also induce additional CGRP release and ultimately evoke increased production of inflammatory mediators which then sensitize trigeminal ganglion neurons further (77, 78) (Figure 2). CGRP secretion from the trigeminal ganglion likely regulates sensory processing (79), and evokes peripheral vasodilation by acting on CGRP receptors on smooth muscle cells of the meningeal vasculature, leading to the release of other neuropeptides that together mediate meningeal neurogenic inflammation (80, 81). Although the precise mechanisms of CGRP as a neuromodulator are not well-understood, CGRP release from unmyelinated C-fibers has been found to directly activate Aδ-fibers which transmit noxious information (82). Additionally, it has been shown that CGRP released in the SpV by trigeminal afferents facilitates nociceptive transmission, which is supported by reduced noxious-related c-fos expression (an index of neural activation) in SpV following CGRP inhibition (83, 84). Further, the co-localization and co-release of CGRP with substance P, a potent modulator of nociceptive transmission in the dorsal horn of the spinal cord has been shown to contribute to pain-related behaviors (85, 86). These reports indicate that CGRP and its receptors have a facilitatory role in modulating the transmission of nociceptive signals to higher cortical brain regions.

Furthermore, sensitization of the ascending trigeminal pathways within the brain itself is likely involved in chronic migraine. Such central sensitization is thought to underpin the presence of cutaneous allodynia in chronic migraine that is more common and severe than that seen in individuals with episodic migraine (87). Interestingly, chronic migraineurs express both cephalic and extracephalic allodynia, suggestive of sensitization of neurons in SpV, where noxious afferents innervating the head and oral cavity first terminate, as well as in higher brain regions such as the thalamus which contains representations of noxious inputs from the entire body (48, 88, 89). Such central sensitization may also underlie higher attack frequency and pain intensity, which are risk factors for the development of cutaneous allodynia in migraineurs (87, 90). Cutaneous allodynia may also underpin migraine chronification and as a consequence, the prevention or reversal of central sensitization may therefore reduce migraine pain and the rate of chronic migraine transformation (91). In conjunction with observations of reduced pain thresholds in chronic migraineurs compared with episodic migraineurs, it is apparent that altered central processing of noxious information (92) may contribute significantly to prolonged pain and hypersensitivity in chronic migraine.

### Functional Brain Alterations in Chronic Migraine

In addition to central sensitization, it is likely that the activation and sensitization of the trigeminal pathway and related pain circuits within the brain become persistent with disease chronification. This may further contribute to the structural and functional reorganization of pain-related circuits in chronic migraineurs, increasing susceptibility to the development of more frequent attacks, thus bypassing the interictal phase in most instances (93, 94).

#### Brainstem

Nerves innervating cerebral vascular and dura enter the brain via the trigeminal nerve and terminate within the SpV. Preclinical studies have shown that these fibers innervate an extensive rostrocaudal extent of the SpV from the caudal extent of the SpV caudalis and cervical 2/3 dorsal horns, rostrally to parts of the interpolaris and oralis SpV subdivisions (95, 96). Second order neurons then contact multiple sites in the brainstem including regions known to modulate incoming noxious inputs such as the PAG. The PAG contains longitudinal oriented columns that when activated produce active and passive defensive behaviors combined with a powerful analgesia (97). Indeed, the PAG and its projection to the rostral ventromedial medulla (RVM) forms the backbone of the brain's powerful pain modulatory system which can increase or decrease the ability of projection neurons in the SpV to activate the cortex and alter the intensity of perceived pain (98–102). Indeed, preclinical studies have reported that electrical activation of the ventrolateral PAG (vlPAG) inhibits nociceptive activity in the SpV following electrical stimulation of the dura mater (103). It has been proposed that dysregulation of the RVM is involved in the initiation of migraine (27) and that modulation of the PAG-RVM-SpV pathway can either initiate a migraine by enhancing basal ascending noxious activity or alternatively provide an environment by which a trigger can evoke a migraine headache (31, 104).

A role for the PAG in migraine headache generation has long been argued, although its precise role in chronic migraine pathophysiology is yet to be fully elucidated. Some have suggested that the PAG may act as a “generator” of migraine attacks, a position supported by a report that immediately following electrode implantation into the PAG, migraine-like headaches occurred in a small proportion of episodic migraine subjects (105). Furthermore, human neuroimaging has revealed activation of brainstem regions including the PAG during spontaneous migraine attacks, with PAG activation persisting even following the alleviation of migraine symptoms by sumatriptan (106). It has also been reported that chronic migraineurs display reduced pain modulatory ability during the interictal period (107) and whilst the function of brainstem pain modulating circuits has not been directly explored in chronic migraine, recent episodic migraine studies reported altered resting state functional magnetic resonance imaging (fMRI) connectivity within the PAG-RVM-SpV pathway during the interictal period (108). Importantly, whilst functioning of this brainstem circuit in chronic migraine is yet to be explored, resting state fMRI connectivities between pain processing regions such as the anterior insula, amygdala and PAG are altered during the interictal period in chronic migraineurs (109) and these alterations are more severe when compared to those in episodic migraineurs (110).

While existing evidence from episodic migraine studies indicates that the PAG may play a distinctive role in migraine pathogenesis (106, 111), further investigations are necessary to delineate whether the PAG is involved specifically in migraine or more generally in pain conditions. Nonetheless, these progressive episodic migraine studies have paved the way for chronic migraine research by delineating the potential role of the PAG in underlying mechanisms of chronic migraine.

#### Hypothalamus

In addition to altered brainstem function, diencephalic changes have been reported in both chronic and episodic migraineurs. Compared to interictal episodic migraineurs, increased functional connectivity between the anterior hypothalamus and SpV was demonstrated in medication-overuse chronic migraineurs scanned during a headache (112). Similarly, chronic migraineurs, majority of whom were scanned during headache, exhibited stronger activation of the anterior hypothalamus in response to painful trigeminal stimulation (113) and SpV in response to visual stimuli when compared to controls (114). These findings suggest that pronounced functional alterations in the anterior hypothalamus may play a role in migraine attack generation and chronification (113).

Whilst there are only a few studies outlining changes within the hypothalamus in chronic migraineurs, numerous investigations have reported hypothalamic changes in episodic migraine. For example, Maniyar et al. reported significant increases in lateral hypothalamic blood flow during the premonitory phase, triggered by intravenous administration of nitroglycerin (115), whereas Meylakh et al. recently reported lateral hypothalamic blood flow decreases immediately prior to a *spontaneously* occurring migraine (116). Furthermore, these blood flow decreases were coupled to decreases in lateral hypothalamic resting connectivity with the PAG, dorsomedial pons and SpV. The hypothalamus also displays altered resting activity patterns immediately prior to a migraine (76) and greater activation during noxious stimulation in episodic and chronic migraineurs during the ictal phase compared to controls (113). Tract tracing investigations have shown that the vlPAG receives projections from lateral hypothalamic regions (99, 100, 117) and stimulation of the lateral hypothalamus can produce analgesia (118). Hypothalamic connectivity during the ictal phase demonstrated enhanced functional coupling with the dorsal pons in comparison to the 3 days prior to headache onset which suggests a potential mechanism for migraine pain sustainment (119). Following trigeminal nociception, hypothalamic activation was recently found to be represented by the 48 h preceding a migraine headache (33).

We have previously shown that changes in infra-slow oscillatory activity within the hypothalamus-PAG-RVM-SpV immediately prior to an episodic migraine may result from increases in transient astrocyte synaptic modulation since calcium waves in activated astrocytes oscillate at similar infra-slow frequencies (120, 121). It is possible that the combination of transient astrocyte activation in addition to altered decreasing modulation by the lateral hypothalamus, results in altered orofacial pain circuitry firing that either generates head pain itself from altered basal firing or alternatively allows for an external trigger to generate head pain. Furthermore, individuals with chronic migraine, majority of whom had insomnia, were found to have altered circadian hormone secretions (122). This included decreased nocturnal prolactin peak, delayed melatonin peak and increased cortisol levels, suggesting that chronic migraine is associated with abnormal patterns of hypothalamic hormonal secretion (122).

#### Cerebral Cortex

In addition to altered pain modulating ability, it has been proposed that chronic migraine is underpinned by an increase in cortical excitability (48, 123). Consistent with this hypothesis, recent resting state fMRI studies found that chronic migraineurs exhibit altered coherence of multiple major intrinsic brain networks including the salience, central executive, dorsal attention and default mode networks (124, 125). Greater headache severity was also found to be associated with increased connectivity strength of the dorsal attention system and lower strength of the executive control network, suggesting that chronic migraine is characterized by maladaptive cortical network plasticity alterations (125). Additionally, chronic migraineurs demonstrate a persistent excitability pattern of the visual cortex during the interictal period, similar to patterns observed during the ictal phase (126) and greater visual cortical excitability (107). In a subset of chronic migraineurs, this change was accompanied by the pons and right temporal cortex activation and the inhibition of cortical areas such as the medial frontal, parietal cortex and somatosensory area (107). These findings infer that a dysfunction in inhibitory capacity may render chronic migraineurs particularly susceptible to increased attacks (126, 127).

These observations in functional changes suggest that chronic migraineurs exhibit aberrant pain processing due to altered descending pain modulation (107, 111). Determining if underlying neural alterations are analogous to both episodic and chronic migraineurs or specifically involved in migraine chronification remains elusive and requires further research (54).

### Structural Brain Alterations in Chronic Migraine

**Figure 3:**
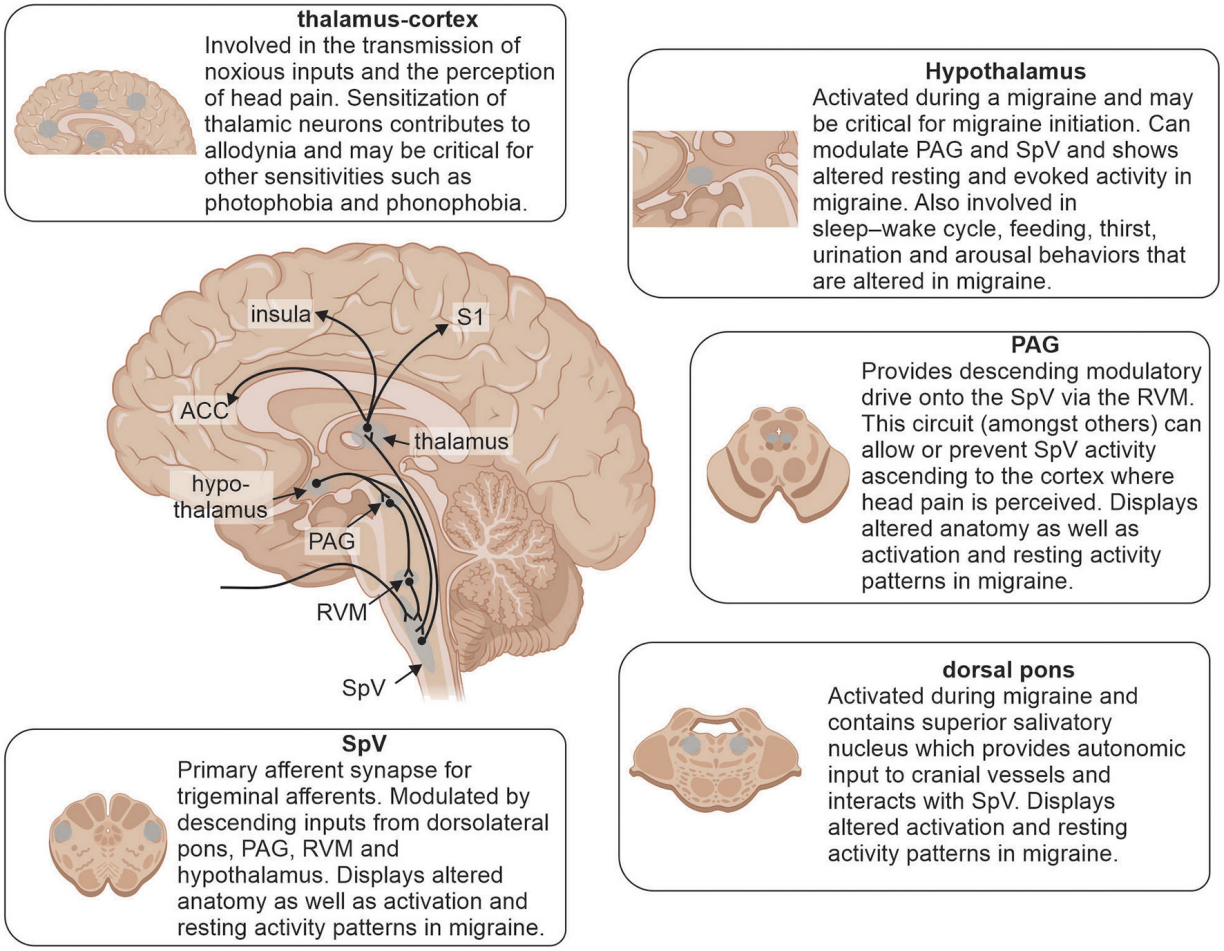
Brain regions involved in underlying mechanisms of migraine and reported alterations. Modulation of incoming noxious inputs: spinal trigeminal nucleus (SpV), periaqueductal gray matter (PAG), rostral ventromedial medulla (RVM) and dorsal pons. Higher order processing: hypothalamus, thalamus, anterior cingulate cortex (ACC), insula and primary somatosensory cortex (S1).

Coupled to changes in brain function, a number of studies have detailed structural changes in individuals with episodic and chronic migraine (Figure 3). Gray matter volumetric changes in several brain regions involved in pain processing including increased gray matter volumes in the amygdala, putamen, PAG and dorsolateral prefrontal cortex as well as decreased volumes in the anterior cingulate cortex, temporal and occipital lobes, precuneus, cerebellum and brainstem have been reported in chronic migraineurs (42, 93, 128, 129). A recent study also found that both chronic and episodic migraine were associated with significantly decreased hypothalamic volumes compared to controls and the reduction in chronic migraineurs was positively correlated with headache frequency (130). These findings are consistent with the idea that the hypothalamus plays a role in migraine chronification potentially through its multiple ascending and descending connections. Furthermore, chronic migraineurs display increased iron accumulation in the PAG and red nucleus (131), which may also result from progressive changes during migraine chronification (132).

Brain structural changes have also been assessed in chronic migraineurs using diffusion tensor imaging. A recent study found that chronic migraine displayed reduced mean diffusivity, an index of white matter integrity, compared with episodic migraineurs in a number of brain regions, in particular brainstem regions encompassing the SpV tract and midbrain, as well as the thalamus, visual and auditory regions (133). This study indicates that brainstem structural changes parallel functional brain changes and may underlie alterations in functioning of the descending modulatory pain pathway in chronic migraineurs. Interestingly, it was recently reported that mean diffusivity within the brainstem fluctuates across the migraine cycle in individuals with episodic migraine (134). More specifically, mean diffusivity was decreased in the area encompassing the PAG, dorsolateral pons and SpV during the 24 h prior to a migraine. These findings are consistent with the idea that immediately prior to a migraine, astrocytes are activated and increase in size within the PAG and SpV, resulting in a decreased free-water movement (diffusivity). This is coupled with an increase in infra-slow oscillatory gliotransmitter release, a subsequent increase in infra-slow oscillatory resting activity and a sensitization of brainstem pain modulatory pathways. Whilst this is entirely speculative, if true, it would provide a new target for therapy development in individuals with episodic and potentially chronic migraine.

## Preclinical Studies of Chronic Migraine

Although advancements in understanding chronic migraine pathophysiology and the development of novel therapeutics can potentially be achieved through preclinical studies, current animal models of chronic migraine remain limited. There are currently several techniques designed to induce headache-like pain in rodents, however, due to the complex nature of migraine, replication of the entire chronic migraine condition remains elusive. Since headache frequency is the key phenotypic difference of chronic migraine, repeated dural applications of “inflammatory soup,” intravenous infusions of glyceryl trinitrate and repetitive administration of acute migraine abortive treatments such as triptans to stimulate medication overuse headache have been most widely implemented to model chronic migraine (135).

Whilst the administration of glyceryl trinitrate in migraine patients (136, 137) has been shown to induce pain, in rodents, glyceryl trinitrate evokes acute hyperalgesia and the development of progressive basal hypersensitivity to mechanical stimulation (138–140). Migraine preventative medications, topiramate and propranolol, have been found to inhibit both acute hyperalgesia and basal hypersensitivity, whereas inhibition of only hyperalgesia was demonstrated with sumatriptan (138–140). The inflammatory soup model, characterized by inflammatory mediators such as prostaglandin and histamine, produced allodynia and spontaneously increased nociceptive behavior in rats which was reduced by zolmitriptan (135, 141). However, the use of chemicals in this technique may compromise blood brain barrier functionality, resulting in the direct activation of central brain sites rather than synaptic transmissions via the activation of meningeal afferent fibers (135). Furthermore, the treatment of migraine through the frequent use of triptans may lead to medication overuse headache and has been identified as a major risk for migraine chronification. The medication overuse headache rodent model encompassing the repeated administration of drugs such as triptans and opioids, was found to elicit a persistent state of latent sensitization (142). This model, however, may unequivocally replicate chronic migraine due to the unlikely occurrence of migraine development following medication overuse in sufferers without pre-existing headaches (94). While no animal model has encapsulated the multifactorial clinical features of chronic migraine, the refinement of existing models and the discovery of novel preclinical models would likely lead to a much greater understanding of the underlying pathophysiology and may facilitate the development of new therapeutic strategies for chronic migraine.

Evolving preclinical models of migraine with potential implications for chronic migraine include the combination of novel targeted optogenetic or chemogenetic approaches to evoke migraine related pain and associated phenotypes (143). In addition, innovative tracing technologies allow detailed mapping of neuronal projections throughout the nervous system (143). Despite the lack of preclinical models of chronic migraine, novel insights into clinical features of this disorder may be explored in non-pain phenotype preclinical models. Clinical obesity is known as a corresponding risk factor for migraine transformation and shares several homogenous pathophysiological mechanisms with chronic migraine (144). Elevated levels of proinflammatory mediators implicated in migraine mechanisms such as CGRP are also observed in obesity (144), suggesting that the migraine-obesity relationship may aid in clarifying underlying mechanisms of migraine applications and reflect the evolving nature of chronic migraine modeling.

## Chronic Migraine Treatments

The development of chronic migraine represents a therapeutic challenge due to poor treatment response with chronic migraine sufferers reporting least satisfaction with primary care relating to treatment (38% compared to 66% of episodic migraineurs) (145). Optimal treatment strategies for chronic migraine encompass risk factor modification, identification of triggers, comorbidity management and the use of acute and prophylactic pharmacotherapy that abort or act as a preventative to a migraine attack, respectively (2, 49, 146). Chronic migraineurs invariably require prophylactic treatment (2) and the incorporation of an effective prophylactic regimen may reduce attack frequency, severity and associated disability to decrease reliance on acute treatment contributing to medication overuse headache (49, 146).

### Acute Treatments

Acute therapies for chronic migraine are essentially those used to treat episodic migraine and include analgesics, non-steroidal anti-inflammatory drugs (NSAIDs) or migraine specific agents with vasoconstrictive properties such as triptans and ergot derivatives (8). Although appropriate acute treatment may assist in the reduction of immediate head pain, the effectiveness and optimization of acute treatment options for chronic migraine are quite limited (147). Importantly, it has been reported that chronic migraineurs exhibit a less robust response to triptans (148) and the addition of further triptans or NSAIDs to an existing triptan based regimen is not associated with improvements in chronic migraine associated disability, as assessed by the Migraine Disability Assessment Scale (149). Furthermore, combination treatment of sumatriptan plus naproxen was found to be effective in acute episodic migraine treatment compared with either monotherapy (150), demonstrating differential treatment between episodic and chronic migraine.

Opioid and barbiturate containing medications are not recommended due to their strong association with medication overuse headache development and medication dependency (18). Due to medication overuse headache propensity and general exacerbation to migraine symptoms if overused, acute treatment should be limited to 2 days per week (151). Monitoring and limiting the use of acute compounds for the treatment of chronic migraine is critical to the avoidance of implicated risks, such as medication overuse headache, which underscores the importance of implementing prophylactic treatments to reduce headache frequency and quality of life parameters (8).

### Prophylactic Treatments

With limited clinical prophylactic treatment options, there remains an unmet need for more effective, tolerable preventative therapeutic targets in chronic migraine (151). To date, the only currently available pharmacotherapies that have demonstrated efficacy in chronic migraine prophylaxis are onabotulinumtoxinA (BoNT-A), topiramate (152, 153) and newly approved CGRP targeted monoclonal antibodies (154, 155).

BoNT-A has been proven to be an effective, safe and well-tolerated treatment for chronic migraine prevention in randomized, double-blind, placebo-controlled studies (153, 156, 157). In these studies, BoNT-A treatment was associated with significantly reduced headache frequency and disability, and improved quality of life irrespective of acute medication overuse (158). It is proposed that injection of BoNT-A inhibits the release of several neurotransmitters such as CGRP, substance P and glutamate from peripheral trigeminal nociceptive neurons and disrupts TRP channels, thereby reducing neuronal hyperexcitability and peripheral and central sensitization (159, 160).

Orally administered topiramate was found to be well-tolerated and reduced the mean monthly headache days in chronic migraineurs compared to placebo in randomized placebo controlled trials (161, 162). Paralleling BoNT-A, topiramate was reported to be effective in medication overuse headache without the removal of overused medications (161). Topiramate may exert its effect in chronic migraine prevention through the reduction of nociceptive transmission via trigeminovascular modulation, which inhibits neuronal hyperexcitability and suppresses the initiation and development of cortical spreading depression (163).

Recently developed as the first fully humanized monoclonal antibody treatments specifically for chronic migraine, fremanezumab (164), galcanezumab (165) and eptinezumab (166) target the CGRP ligand, while erenumab (167) targets the CGRP receptor. These novel antibody treatments are postulated to neutralize the effects of excessive CGRP released in the trigeminal sensory nerve fibers during migraine attacks (168). Each of these anti-CGRP monoclonal antibodies have been proven effective, tolerable and safe as chronic migraine prophylactic treatments in clinical trials (155, 167–170). Eptinezumab is the first intravenous anti-CGRP monoclonal antibody treatment in chronic migraine, while fremanezumab, galcanezumab and erenumab are administered subcutaneously (171, 172). Through bypassing the liver, these anti-CGRP therapeutic agents overcome hepatotoxicity risks and central nervous system related effects associated with CGRP receptor antagonistic molecules (173). Treatment outcomes are greatly improved with the use of novel anti-CGRP monoclonal antibody treatments and they were reported to significantly reduce mean monthly headache days (164, 165, 167, 174) with combined preventative therapy (175), providing further support to established conventional therapeutics in chronic migraine prophylaxis.

Additional chronic migraine prophylactic agents shown to be effective in limited studies include valproate, gabapentin, pregabalin, tizanidine, zonisamide and amitriptyline (176–181). Further evidence obtained through randomized clinical trials are necessary to demonstrate their efficacy in chronic migraine prevention.

### Non-pharmacological Emerging Treatments

A promising emerging treatment for pharmacologically non-responsive or intractable chronic migraine is neuromodulation. Demonstrating promising results, non-invasive neurostimulation modalities include supraorbital transcutaneous stimulation, transcranial magnetic stimulation, transcranial direct current stimulation and non-invasive vagus nerve stimulation (182–184). Invasive methods include implanted vagus nerve stimulation, occipital nerve stimulation, sphenopalatine ganglion stimulation and deep brain stimulation (185–188). While recent technological developments in neuromodulatory methods have presented greater opportunity for the successful treatment of chronic migraine, further evidence-based randomized controlled trials are required to assess long-term safety and efficacy before they are established as standard treatments.

## Future Developments

Whilst there has been considerable preclinical and human work exploring the mechanisms responsible for migraine, particularly migraine headaches, only a handful of studies have explored the pathophysiology of chronic migraine. Given that chronic migraine undoubtedly involves altered function of brainstem and hypothalamic nuclei, the limited spatial resolution obtained using high field MRI scanners, has meant that accurate and robust exploration of these areas in humans has been a significant challenge. The relatively recent advance in MRI field strength, for example the development of 7 Tesla (7T) human MRI machines means that we are now able to explore structures in the order of 1 mm^3^ which will allow us to explore the brainstem and hypothalamus in chronic migraineurs in detail. The addition of other measures exploring psychological state, peripheral immune, genetic and sleep measures in combination with high-resolution MRI will provide a more global view of chronic migraine and will pave the way for a greater understanding of the underlying pathophysiology. While 7T MRI offers increased signal-to-noise-ratio, there is a significant associated cost and limited availability, coupled with current technical limitations, such as enhanced susceptibility to image artifacts and contrast changes (189, 190). Further limitations include prolonged T1 relaxation times and increased radiofrequency (RF) energy deposition in tissue, limiting some scan sequences (191). Despite these technical challenges, evolving research continues to address and improve 7T clinical outcomes by optimizing and customizing sequence selection in addition to technological advancements such as parallel transmit coils and processes to minimize RF energy (189–191).

In addition to significant advances in technical capabilities, determining why some individuals respond to particular treatment regimen and others do not may provide vital information about individual differences and potentially drive more personalized treatment options. It is known that CGRP inhibitors are effective in reducing chronic migraine frequency in only a proportion of individuals, and why this is the case remains unknown. Whilst it is evident that anti-CGRP monoclonal antibodies are efficacious in treating chronic migraine with medication overuse, longitudinal neuroimaging studies are warranted to assess structural and functional changes. Exploring the effectiveness of current treatments such as CGRP inhibitors on migraine frequency and brain function, could provide us with details of differences in central and peripheral mechanisms between individual chronic migraineurs and evidence of the balance between peripheral/central mechanisms in chronic migraine. Such an approach could lead to an understanding of individual differences in the pathophysiology of chronic migraine, a biomarker for subsequent CGRP treatment efficacy and the development of personalized treatment regimens. Only by understanding the pathophysiology of chronic migraine and individual variations are more effective treatment regimens going to be developed.

## Conclusion

Chronic migraine is a highly debilitative disorder with significant individual, economic and societal burdens. The pathophysiological mechanisms of chronic migraine and its progression from episodic migraine remain poorly understood. Dysfunction of the descending pain modulatory pathway, enhanced cortical hyperexcitability, central sensitization and structural brain changes have been proposed as important considerations in the development of chronic migraine. Despite our limited understanding about the underlying pathogenesis of this condition, it is conceivable to infer that chronic migraineurs are in a constant premonitory phase which may perpetuate abnormalities in the descending pain modulatory pathway, cortical hyperexcitability and central sensitization. While topiramate, BoNT-A and recently approved anti-CGRP monoclonal antibodies are currently the only efficacious therapeutics in chronic migraine prophylaxis, neurostimulation creates new and promising outcomes in chronic migraine management. Future research is warranted to determine if effective migraine therapies can prevent or reverse the persistent structural and functional alterations evidenced in chronic migraine. The refinement of preclinical models and advancement of neuroimaging techniques may facilitate the discovery of new, individualized therapeutic targets and provide a greater understanding of the complex, multifaceted condition that is chronic migraine.

## Author Contributions

TM wrote the original draft and final version. LH and NM helped write each version of the manuscript following the initial draft. All authors contributed to the article and approved the submitted version.

## Conflict of Interest

The authors declare that the research was conducted in the absence of any commercial or financial relationships that could be construed as a potential conflict of interest.

## Publisher's Note

All claims expressed in this article are solely those of the authors and do not necessarily represent those of their affiliated organizations, or those of the publisher, the editors and the reviewers. Any product that may be evaluated in this article, or claim that may be made by its manufacturer, is not guaranteed or endorsed by the publisher.
